# CMT Additive Manufacturing Parameters Defining Aluminium Alloy Object Geometry and Mechanical Properties

**DOI:** 10.3390/ma14061545

**Published:** 2021-03-22

**Authors:** Gyula Ferenc Vasvári, Dávid Csonka, Tamás Zsebe, Ádám Schiffer, Ivan Samardžić, Roland Told, Attila Péntek, Péter Maróti

**Affiliations:** 1 Department of Mechanical Engineering, Faculty of Engineering and Information Technology, University of Pécs, Boszorkány Street 2, 7624 Pecs, Hungary; vasvari.gyula@mik.pte.hu (G.F.V.); schiffer.adam@mik.pte.hu (D.C.); tamas.zsebe@mik.pte.hu (T.Z.); 2 Faculty of Engineering and Information Technology, Institute of Information and Electrical Technology, University of Pécs, Boszorkány Street 2, 7624 Pecs, Hungary; adam.schiffer@mik.pte.hu; 3 Department of Technology, Faculty of Mechanical Engineering, University of Slavonski Brod, Gundulića 20A, 35000 Slavonski Brod, Croatia; ivan.samardzic@sfsb.hr; 4 Medical Simulation Education Center, Medical School, University of Pecs, Szigeti Street 12, 7624 Pécs, Hungary; told.roland@pte.hu (R.T.); pentek.attila@pte.hu (A.P.)

**Keywords:** additive manufacturing, cold metal transfer, 3D printing, geometrical analysis, aluminium, mechanical testing

## Abstract

Additive manufacturing technologies based on metal melting use materials mainly in powder or wire form. This study focuses on developing a metal 3D printing process based on cold metal transfer (CMT) welding technology, in order to achieve enhanced productivity. Aluminium alloy test specimens have been fabricated using a special 3D printing technology. The probes were investigated to find correlation between the welding parameters and geometric quality. Geometric measurements and tensile strength experiments were performed to determine the appropriate welding parameters for reliable printing. The tensile strength of the product does not differ significantly from the raw material. Above 60 mm height, the wall thickness is relatively constant due to the thermal balance of the welding environment. The results suggest that there might be a connection between the welding parameters and the printing accuracy. It is demonstrated that the deviation of ideal geometry will be the smallest at the maximum reliable welding torch movement speed, while printing larger specimens. As a conclusion, it can be stated that CMT-based additive manufacturing can be a reliable, cost-effective and rapid 3D printing technology with enhanced productivity, but without significant decrease in mechanical stability.

## 1. Introduction

In the last decade, an increasing number of metallic 3D printed parts used in the machine production industry [1] and metal base additive manufacturing (AM) has also emerges in medical applications [2]. Metallic additive manufacturing is widely used in rapid prototyping and in small series production, being capable of printing complex geometries. However, it is important to note that the production of simple parts with the commercially available 3D metal printers is slower compared to traditional manufacturing technologies like turning and milling [3]. Thereby, it might be more expensive to produce a part with less complex surfaces.

The metal additive manufacturing technologies are based on metal melting or welding processes with highly diverse technical solutions [4]. There is a wide variety of AM equipment where metal powder is melted by laser or electron beam. The most widely used metal printing method in the machine industry and healthcare is direct metal laser sintering (DMLS) technology. The procedure is similar to selective laser sintering (SLS) [5]. In this process, a laser beam scans the layer, melts and welds the metal powder into the layer beneath. in electron beam technology (EBM) the metal powder layer is melted by a magnetic leaded electron beam in a vacuum chamber. It is a popular technology as well, because of the fact, that deeper melting can be achieved with high kinetic energy electrons; thus, a thicker layer can be made in one step. The product resolution of these processes is determined by the diameter of the beam and the layer thickness [6]. Thus, these are excellent for the production of detailed metal parts with complex geometry, small size and high dimensional accuracy using various metallic base materials as titanium, aluminium alloys or special purpose steel. However, serious disadvantages are present, like relatively high investment and maintenance prices [7]. In addition, handling highly explosive metal powders requires special attention and safety precautions [8]. In Table 1 a detailed comparison is made using data and calculation formulas of Laureijs et al. [7]. The data were reconstructed from the Supplementary Material of the previous work of Laureijs et al. [7] and extended by our own findings.

As metal additive manufacturing is a novel technology, the base for comparison is scarce. In the literature, the cost effectiveness of EBM and DMLS technologies are available; however, there is no economical data on other methods like LMD. Wire and arc additive manufacturing (WAAM) would be best to compare to LMD, but the comparison with EBM and DMLS is also an existing method of cost assessment [9].

Using wire-based technology, it is possible to develop a fast, sufficiently accurate and reliable application that can compete with conventional machining techniques. However, the surface accuracy is lower compared to the powder based metallic 3D printing, the surfaces that require higher accuracy can be post-machined. The wire based additive technologies are based on well-known overlay welding methods [10] by mounting a welding torch on a CNC-controlled equipment. Therefore, in these processes, the resolution of the product is determined by the wire cross section, travel speed, feed rate and the welding parameters [11]. To perform the experiments, we built a metal printer based on cold metal transfer (CMT) welding machine with a unique drive mechanism and control. This is a kind of wire and arc additive manufacturing. The CMT technique has a wide variety of applications, such as cladding, additive manufacturing, composite joint pin fabrication and crack repair utilized in various industries, including automotive and marine [12]. Cold metal transfer is a preferred solution when the aim is to weld different metal types with various thicknesses. To produce characteristics such as low voltage, low heat welding works properly on thin sheet metal. When fusion of thicker materials is needed, integrity of the weld is essential. The CMT process provides a strong weld with good structural properties while keeping the heat input at lower rates, thus resulting in only minimal structural changes due to heat deformation [13]. Thin metal parts have a greater possibility of distorting when heated, during traditional gas metal arc welding (GMAW) heat sinks or other heat protection must be used to prevent the warping of the metal, while heat protection is not needed during the CMT process [14]. In a previous study investigating thin, single bead parts with WAAM technologies found no difference due to rate of heat input, current and voltage ratio on hardness tests and geometrical changes were examined as well [15].

CMT is a modified metal inert gas (MIG) welding technology developed by Fronius International GmbH, (Froniusplatz 1, 4600 Wels, Austria) and is based on short-circuiting transfer process technique. The mechanical droplet deposition method of CMT differs from MIG/MAG welding [16]. Specialities of CMT are the method of material deposition and the low thermal input via high-speed digital control of the wire feed system [17]. Two main features are distinguished, the first is the short circuit with low current corresponding to a low heat input. The second is short circuit occurrence in a stable controlled manner. A previous investigation reported the short circuit transfer process called “mechanically assisted droplet deposition” preferred in controlling when the wire retracted from the short circuit [18]. To reduce the spatter, CMT technology applies droplet detachment mode, while MIG process works with electromagnetic force [13,19].

The primary aim of the research was to find an optimal toolhead movement velocity range where the movement is slow enough to maintain weld arc stability, while rapid enough that the geometric deformation caused by heat is at minimum value. We assume that the highest stabile speed will produce the highest print resolution. This theory has to be validated by geometry measurements. Furthermore, in this study, we attempt to find a relationship between manufacturing parameters and the geometry of overlay welded seams [15,16], alongside with mechanical properties. To determine the structural characteristics of the welded objects, the surface was examined using scanning electron microscopy and optical microscopy as well.

## 2. Materials and Methods

### 2.1. Development of CMT AM Device

**Figure 1:**
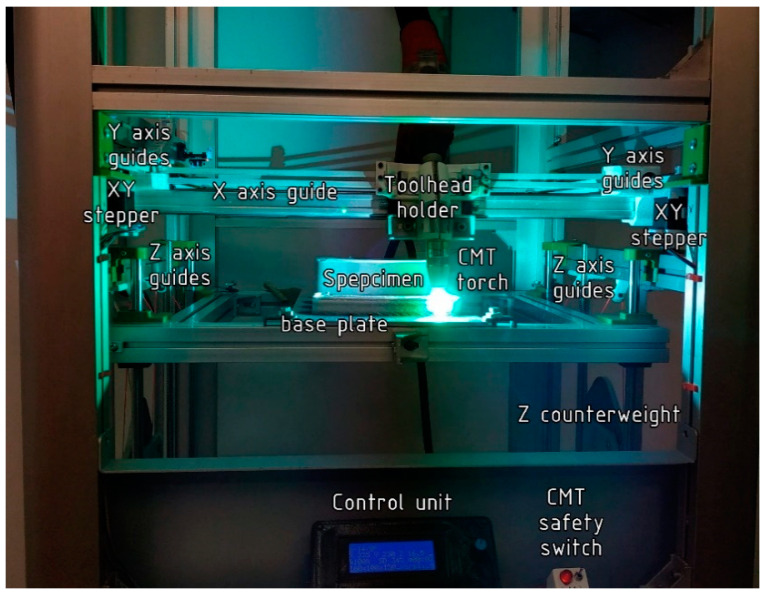
Custom-made CoreXY based cold metal transfer additive manufacturing equipment.

A custom-made 3D printer hardware have been developed by our research team, which performs the control of the welding torch and the printer bed (Figure 1). The actuating mechanism of the equipment is based on HyperCube, an open source 3D printer design. The motion mechanism is CoreXY [20], in which compared to classical Cartesian system, the combined rotations of the motors causes the motion of the head. The whole design is modified in a manner that the structure would be stabile under the load of the welding apparatus, which is heavier compared to FDM printer head. The modified structure leads to the need of redesigning the fittings as well. The FDM printer head is replaced by the CMT welder device (Fronius International GmbH, (Froniusplatz 1, 4600 Wels, Austria); thus, the toolhead mount is designed to be capable of holding the CMT welding torch.

The firmware of the RAMPS 1.4 (RepRap Arduino MEGA Polulu Shield) motherboard was created using Marlin Config software (open source software with GPLv3 license). The major differences compared to a classic HyperCube were the lack of extruding steppers, heating tools and fans. Instead, the switching of the CMT welding device was needed and a cooling fan output was used for this purpose. This is an easily programmable output and the CMT switching could be implemented in the generated gcodes.

### 2.2. Material Properties

Chemical composition of the welding material according to the manufacturer’s certificate is shown in Table 2. These values are checked using SEM spectroscopy. The used equipment is (JEOL JSM-IT500HR, 3-1-2 Musashino, Akishima, Tokyo 196-8558, Japan) The material chemical composition was measured in three separate points.

### 2.3. Settings for Production of Overlay Welded Ribs and Parts

**Figure 2:**
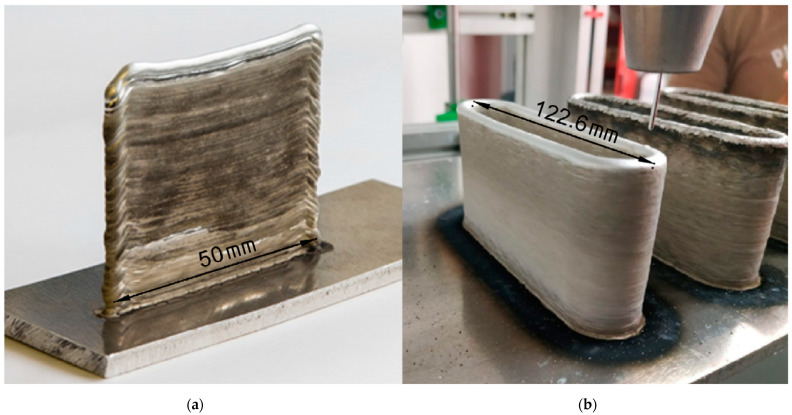
CMT additive manufactured pieces. The scales are in [mm] dimension. (**a**) 55-layer wall; (**b**) 300-layer loop.

As a first step, small size test specimens were fabricated to define the welding parameter threshold for further experiments. In the first experiments, 10 layer high, 50 mm long welded specimens have been printed on the base plate. Different electric current values (50–128 A) and tool movement speeds (30–85 mm/s) were tested. These tests were repeated with higher (max. 30 layer high) specimens. In these experiments arc ignition of every layer caused deformations; thus, to avoid this, closed loop layer specimens were printed. Figure 2, panel (a) demonstrates how edges of a non-loop specimen deforms because of arc ignition.

The next experiment was carried out using a closed loop layer specimen series. Similarly to the first experiment, in this case different settings were tested in order to reveal the reliable threshold of parameters. In the initial layers, we applied higher electric current settings and in higher layers we decreased it to minimize heat input. The parameters of welding are summarized in Table 3. 

The first layer was made using the MIX program of the CMT equipment. This process alternates conventional and CMT pulsed material transitions, pulse by pulse. We gradually decreased current values in the first five layers starting from 110 A and reaching 59 A by the fifth layer, leaving this current value unchanged after. Weld seam protection and welding arc stability was provided by argon-based shielding gas in 15 l/min volume flow. 

After finding the appropriate welding electrical current values, a stabile welding arc was maintained and 300-layer high specimens were printed (Figure 2 panel (b)), with three different length and welding speed values giving nine setting combinations for additional testing, shown in Table 4.

Four of the nine sets of parameters were found to run stabile and without errors. In the other cases the seams tend to break, resulting in faulty bodies. The experiments were continued with the selected four reliable and stabile parameters. The toolhead movement velocity determines welding speed and print time of each layer. Therefore, the toolhead velocity value is one of the most important factors assessed in this study. 

Heat input is another main aspect we aimed to investigate as it is directly affecting geometric distortions. Therefore, we extract heat input values from the welding equipment to assess each set of settings in the aspect of heat input.

### 2.4. Composition and Properties of Base Material

In WAAM processes, the welding wire diameter is usually between 0.2 mm and 1.2 mm [3]. In our method, we used a 1.2 mm diameter aluminiumwire and the welded seam rows are built on a 5 mm thick aluminiumbase plate. Both the wire and the base plate material is W.Nr.: 3.3547, AlMg4.5Mn0.7(EN-AW5083) (distributor: Cooptim Hegesztéstechnikai Kft., Géza u. 54. 8000, Székesfehérvár, Hungary; manufacturer: Böhler Welding Group GmbH, Peter-Müller-Straße 14-14a. 40468 Düsseldorf, Germany). The base plate and wire material is widely used in mechanical engineering [21], shipbuilding [22] and in the chemical industry. Furthermore, it is the most non-heat-treatable aluminiumalloy with high tensile strength (average of 275 MPa), which even has outstanding corrosion resistance properties [23]. It may also be interesting to use this material in metal 3D printing because of its relatively low formability, it could be used to manufacture more complex pieces. This would be relatively difficult to be done by stamping or rolling and its deep drawability also have narrow limits [21,22]. 

### 2.5. Mechanical Tests

**Figure 3:**
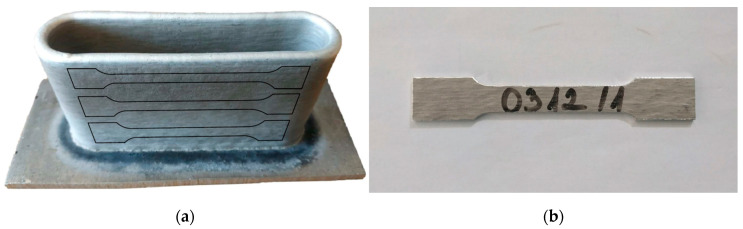
Production of stress test specimens perpendicularly to the weld direction: (**a**) location of specimen on the print; (**b**) machined specimen.

Tensile test specimens were machined by milling technique from the welded loop parts described in 2.2. (Figure 2 panel (b)). From one side of a part, three specimens were made parallel to the direction of seam rows (Figure 3). Tensile tests were performed to determine the effect of welding parameters on tensile strength.

For each of the four sets of settings 11 tensile strength test specimens were machined parallel with the layer direction. The geometry of the tensile strength test samples complied with the EN ISO 6892-1-2016 standard. The tests were carried out using Zwick/Roell Z100THW (sn: 731741/2018) universal materials testing equipment (manufacturer: ZwickRoell, 89079, August-Nagel-Straße 11, Ulm, Germany).

### 2.6. Layer Width Measurements

Every test specimen was cut into two and the thickness was measured every 1 mm on the cut surface. This shows the thickness change along the height of the body. An independent samples t-test was carried out using IBM SPSS 25 to evaluate the difference between the measured layer width values of each sets of settings in neighbouring pairs.

### 2.7. Scanning Electron Microscopy (SEM)

**Figure 4:**
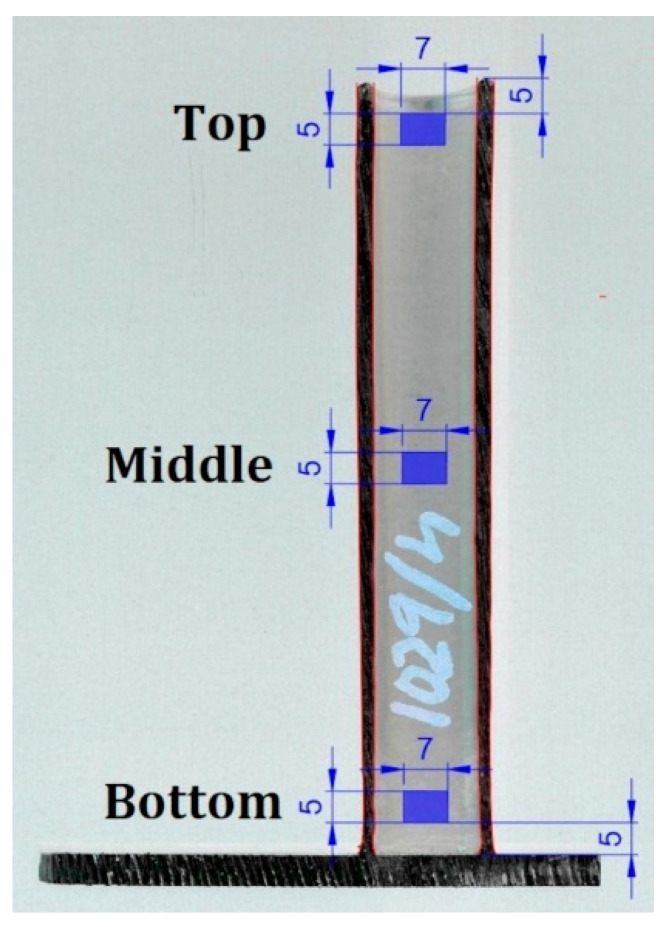
SEM sample locations on the printed part. The units are in [mm].

For SEM (JEOL JSM-IT500HR, 3-1-2 Musashino, Akishima, Tokyo 196-8558, Japan), with 15×–500× magnification. The samples were cut from the 3D printed specimens. A 5 mm ± 1 mm × 7 mm ± 1 mm sample was taken 5 mm from the top, 5 mm from the bottom and from the middle section of the specimens Figure 4 Samples were cut out from 1, 2, 4 and 5 sets of settings. Three samples from one specimen were soldered onto a 30 mm × 50 mm aluminium plate (bottom, middle and top).

Structure analysis of the machined and the tore surfaces was carried out using with the same SEM and magnification, the analysis made with EDS (energy-dispersive X-ray spectroscopy) method with SDD (silicon drift detector) the method of semi-quantitative nature; therefore, the results must be considered as approximate [24].

Material defects were examined using light microscope (Zeiss Primotech KMAT, Carl Zeiss CMP GmbH, 37081, Königsallee 9-21, Göttingen, Germany). Samples were cut from the 3D printed specimens, out from 1, 2, 4 and 5 sets of settings. Bonding zone was examined. Aluminium-oxide (Al_2_O_3_) was used for fine polishing, hydrofluoric acid solution (1 cm^3^ 40% HF + 1.5 cm^3^ HCl + 2 cm^3^ HNO_3_ + 95 cm^3^ distilled water) was used for etching.

## 3. Results

### 3.1. Material Analysis

**Figure 5:**
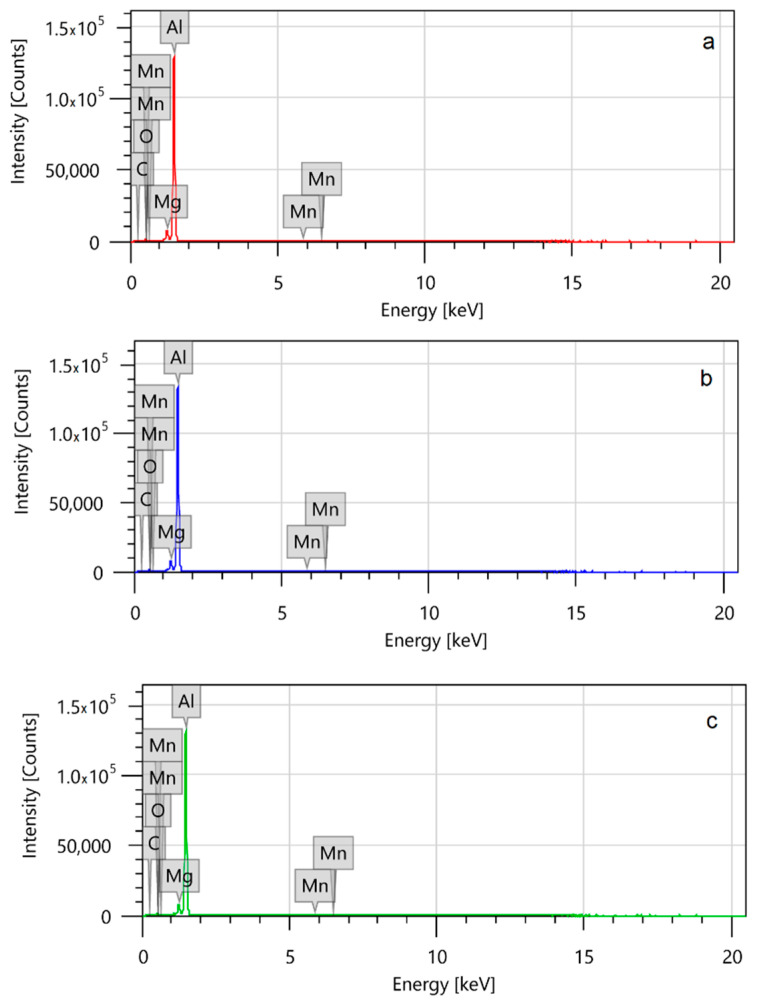
Spectroscopy results of the base material at the three (**a**–**c**) inspection points. Subfigures (**a**–**c**) refer to the three different points where the analysis have been performed on the sample. The results have to be considered to be approximate.

The spectroscopy results shown in Figure 5 and Table 5 verify the required chemical composition of the base material, however, carbon and oxygen are found on the material surface as well. So the atmospheric value of the raw material was calculated (corr. value in Table 5). The EDS-SDD method is not completely reliable; however, it gave a standard value for the raw material in proportions, so it will not be examined further.

### 3.2. Heat Input and Volume Flow

**Figure 6:**
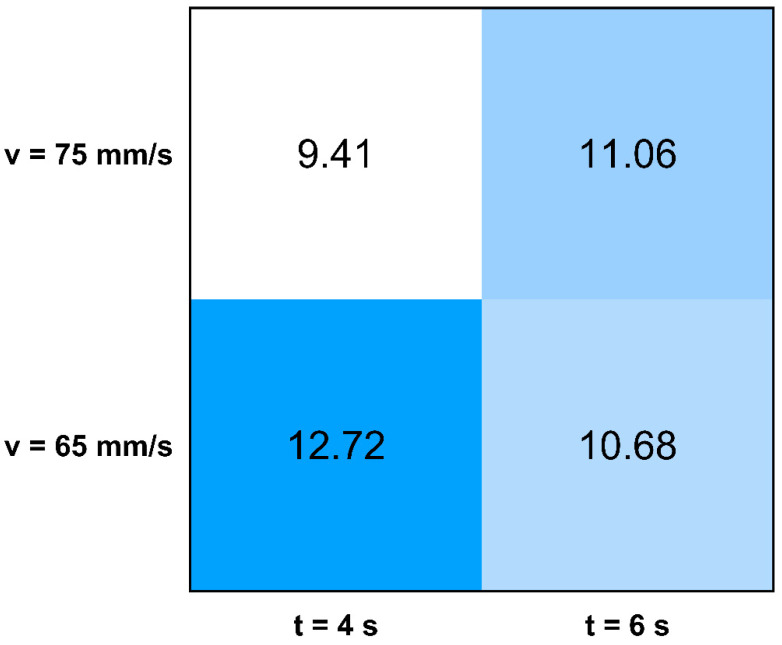
Heat map of the specific heat input in the factor of the manufacturing parameters.

Table 6 and Figure 6 shows total heat input of 300-layer specimens. The heat input values are extracted from the welding equipment and specific heat is calculated by dividing the extracted heat values by the total weld length. Other experiments were carried out using 85 mm/s toolhead speed, but the material deposition was instable and resulted in a discontinuous seam.

### 3.3. Results of the Mechanical Tests

**Figure 7:**
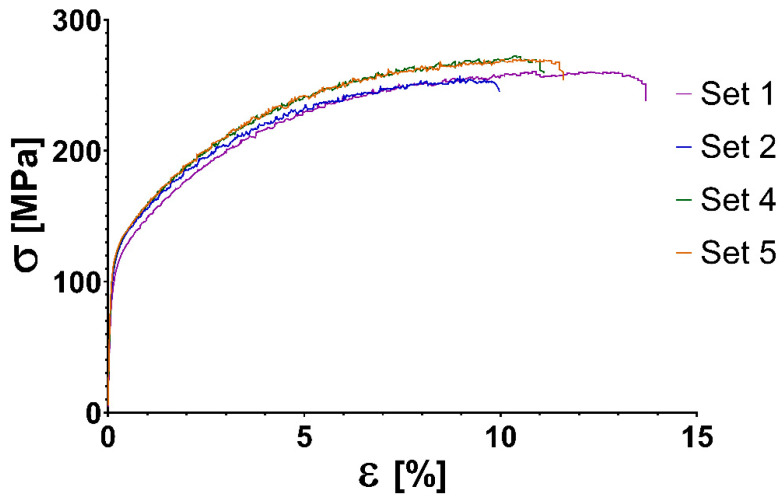
Stress–strain curves per sets of settings of AlMg4.5Mn0.7 3D printed specimens manufactured parallel with the welding direction. σ, stress in MPa; ε, engineering normal strain.

There was no definite yield phenomenon on the tensile diagrams shown in Figure 7 as it was expected for aluminium materials.

**Figure 8:**
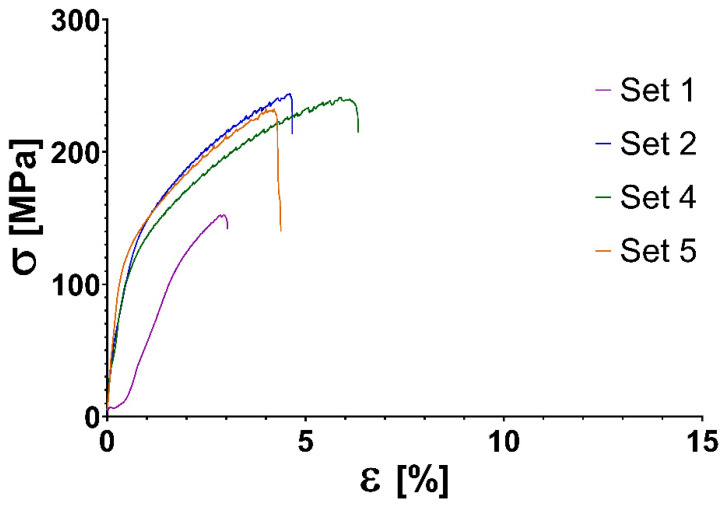
Stress–strain curves per sets of settings of AlMg4.5Mn0.7 3D printed specimens manufactured perpendicular to the welding direction. σ, stress in MPa; ε, engineering normal strain.

The elongation is significantly lower in the case of the specimen manufactured perpendicular to the welding direction compared to the parallel one, as seen on Figure 8.

Based on the results of the tensile tests shown in Table 7, it can be stated that the tensile strength of the specimens parallel with the weld direction does not show significant difference (1.8–11.6%) from strength of raw material (275 MPa). The results show that sets 4 and 5 have somewhat higher tensile strength parallel with the weld direction than sets 1 and 2 (difference: 2–11%).

As shown in Table 8, in set 1., tensile strength is lower in the case of the specimen manufactured perpendicular to the welding direction compared to the parallel one. The tensile strength is only 57% of the base material. In the case of other sets, the tensile strength difference is not significant between the specimen manufactured parallel and perpendicular to the weld direction.

### 3.4. Layer Width Measurements

**Figure 9:**
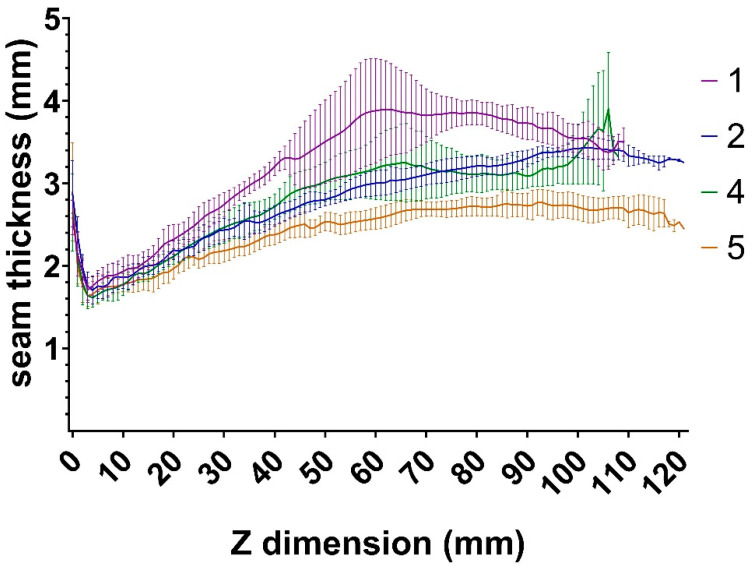
Seam width values per set id. as a factor of z dimension with ±SD bars.

The measurement results show that along the vertical axis the seam width values are increasing. Figure 9 shows the layer width values as a factor of z dimension in the case of the four set of parameters. It is demonstrated in Figure 9 that above 60 mm height within each setting, the seam width fluctuations are lower as the heat input and dissipation are balanced in this region.

At higher speed settings and larger specimen (Set 5), the measurement results show a lower top end thickness and standard deviation, which means higher stability as seen in Figure 9. The t-tests show that the difference between 1–2, 2–4 and 4–5 is significant in 75%, 20% and 84% of the curves, respectively. (*p* < 0.05) If the difference is significant between the neighbouring values, then it also must be significant between curves with a greater difference.

### 3.5. Layer Height Measurements by Scanning Electromicroscopy

**Figure 10:**
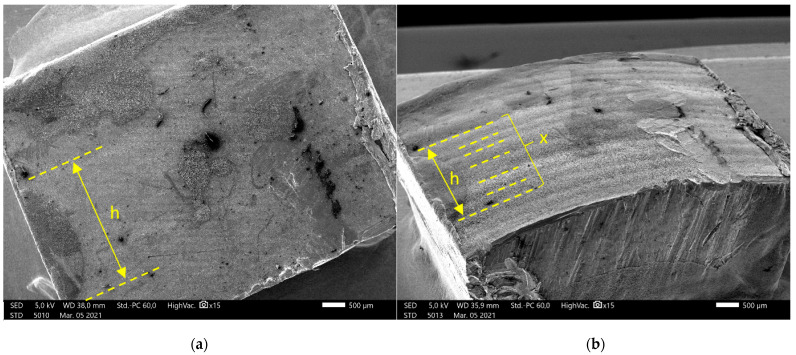
Layer height measurements based on SEM microscopy. h, sample size; x, layer count. (**a**) The image taken perpendicularly, (**b**) the Image from the inclined sample.

In some cases, the layers are not visible on the images taken perpendicularly to the surface (Figure 10 panel (a)); therefore, inclined images were made (Figure 10 panel (b)) and on these it was possible to count the layers (x). By measuring the exact sample size (h) on the perpendicular images, the average layer height could be calculated for these smooth samples.

$$Layer\;height=\frac{h}{x}$$

**Figure 11:**
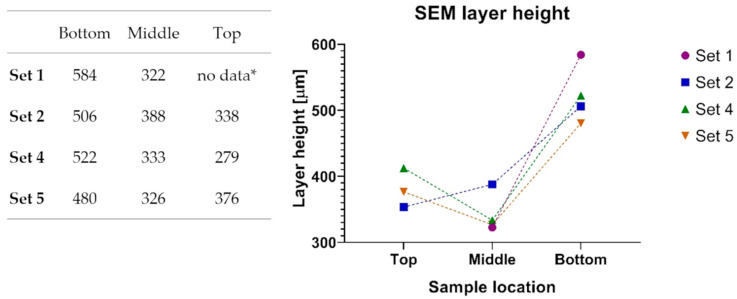
Layer heights based on SEM measurements. * the surface is so smooth; layers are not visible.

The SEM imaging and measurement results show that the layer height is decreasing as expected but surprisingly, in a nonlinear manner. (Figure 11)

### 3.6. Surface Analysis by Microscopy

**Figure 12:**
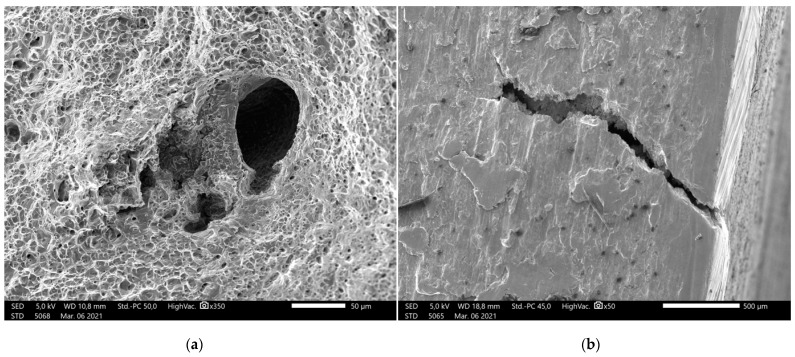
(**a**) SEM image of the torn surface of a tensile strength test specimen with inclusion, (**b**) microcrack in the machined side of specimen.

However, the SEM results shows perfect fusion of the layers and no structural errors on the outer surface of the specimens, it shows minor imperfections inside the material appearing as microcracks or spherical inclusions as seen on Figure 12. Both the torn surface of tensile strength test specimens and the machined side surface of the SEM specimens were inspected and an average of 1–2 inclusions were found in both. These do not appear to significantly affect the tensile strength of the material; however, it might be an explanation for the deviation of the tensile strength test results.

**Figure 13:**
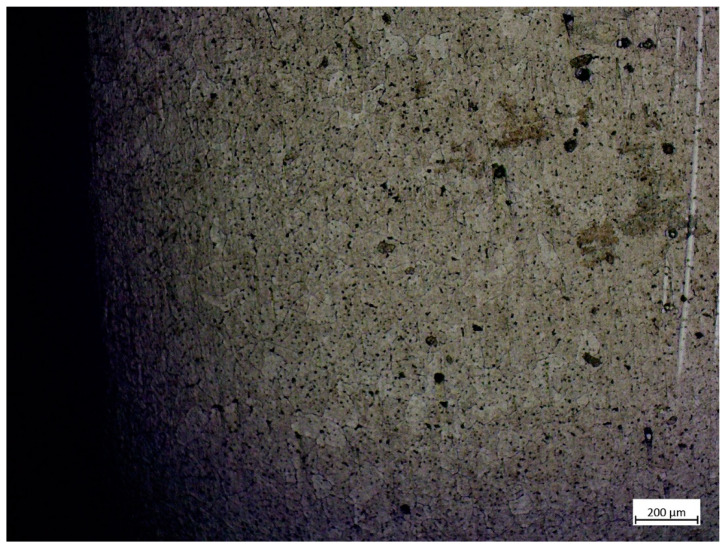
Etched metallography results of the base material using 50× magnification.

The etched metallography results show that the texture boundaries are visible on the images made with 50× magnification (Figure 13) and the deposited material looks homogeneous; the boundary of the bonding zone is not visible.

**Figure 14:**
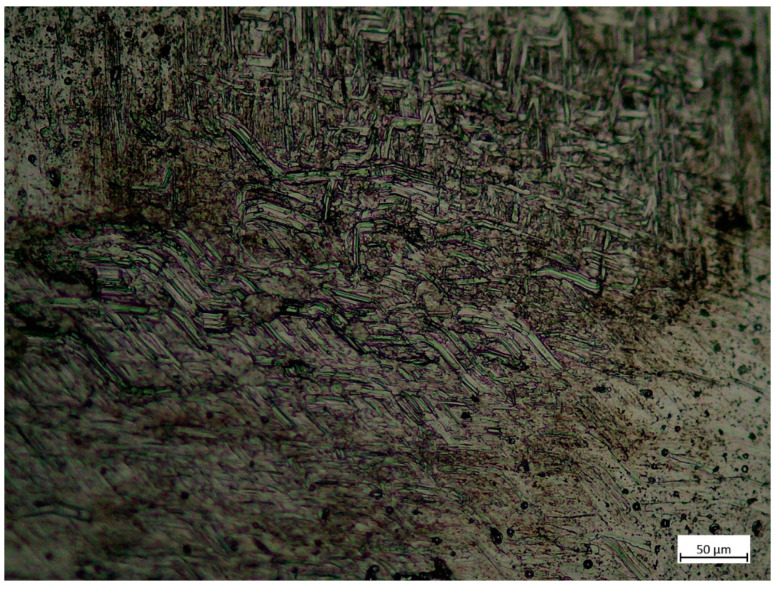
Etched metallography results of the base material using 50× magnification.

At a higher magnification of 200× (Figure 14) oblong crystal texture can be seen. This structure seems ordered in the middle of the image in a line slightly tilted to the right from horizontal. As this direction fits the layer bonding zone direction, the crystal pattern might be the boundary. 

## 4. Discussion

### 4.1. Discussion of the Technological Experiment Results

Based on the results, during overlay welding, the higher layers suffered a higher heat load as the heat dissipation rate is lower with relatively constant heat input. The higher heat load and lower cooling leads to thinner layers in the top half of the specimen compared to the bottom half. This results in a flawed Z directional precision. The results demonstrate that compared to other MAG welding processes, the CMT process has a lower heat input, but the print is adequately accurate only if correct welding parameters are used. We observed that the layer is melted back at the start and end point of welding. This phenomenon made the weld seam wavy. Thus, the distance of welding wire was different along the seam and the geometry became distorted. The best solution for this problem was to print closed loop parts and to use a continuous welding arc during the whole fabricating process. This way, the arc was not eliminated when the torch moved to the next layer. 

We assume that the height dependent stretching of the seam rows might be originating from the higher accumulated heat values at higher layers. The results suggest that heat dissipation decreases as the specimen height increases and the welding position gets farther away from the base plate, which is a large heat-dissipating surface. As the height increases, the heat flux might not be fast enough to carry the heat to the base plate and it accumulates in the last few layers. Because of this, the weldment droplets solidify slower and gravity distorts its shape resulting in the wider weld seam that we experienced. The other geometry issue originating from the abovementioned phenomenon is the inaccuracy of the z dimension. In order to achieve a precise and reliable geometry print, this inaccuracy has to be fixed, which inspires further research projects.

**Figure 15:**
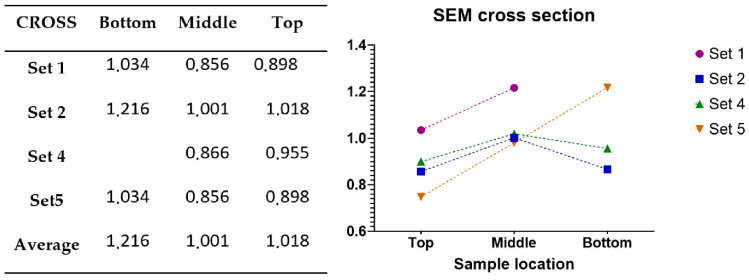
Calculated cross-sectional areas of layers in mm^2^ based on SEM measurements.

The welding parameters are unchanged during the printing of a single specimen, so we assumed that the deposited material quantity is constant; therefore, the layer cross sectional surface should be the same. This theory was not adequately supported by the result of the SEM microscopy results. The cross-sectional surface was calculated from the layer height measurements using SEM imaging and the width measurements in the matching area of the specimens. As shown in Figure 15, the calculations demonstrate that the layer cross sectional surfaces show some change during the printing process. Because of this, further investigation is required to solve the z dimension inaccuracy problem.

We assume that the solution to the z-axis dependent seam width deformation would be to adjust the print speed according to the width curves. This method requires advanced programming of the gcode generation, but we expect it to produce much more precise print geometry.

The heat input results suggest that with shorter layer time (t =4 s) the heat input depends more on toolhead moving speed than in the case of longer layer-time (t = 6 s) specimens (Table 6). At short layer time a slower toolhead movement gives a significantly higher heat input while at longer layer time the difference is negligible.

Volume flow is relatively constant, the results suggest that it does not depend on the welding parameters.

Stress tests show that the material is anisotropic, as perpendicular to the weld direction the samples show a slightly lower tensile strength and significantly lower engineering normal strain. The material AlMg4.5Mn0.7 is not used in laser sintering or casting; therefore, comparison of the stress test with other technologies is difficult. The finding, that tensile strength of the majority of the specimens have 85.8–98.2% the strength of base material (275 MPa) suggests that the technology developed by our team can produce parts with satisfactory strength. It is important to notice that the strength of aluminiumparts manufactured by DMLS according to Martin et al. [25]. compared to the strength of the wrought parts of the same material is only 5.5% in case of Al7075 (T6); 67–73% for Al7075 + Zr (T6) and 67–71% for AlSi10Mg.

### 4.2. Discussion of Video Microsopy and Metallography Results

The microscopy images show minor cracks; these, however, do not seem to affect the strength of the material significantly according to the stress test results.

The metallography results suggest that the bonding of layers is excellent. This property is verified by the stress test results.

### 4.3. Productivity Comparison of Different Processes

**Figure 16:**
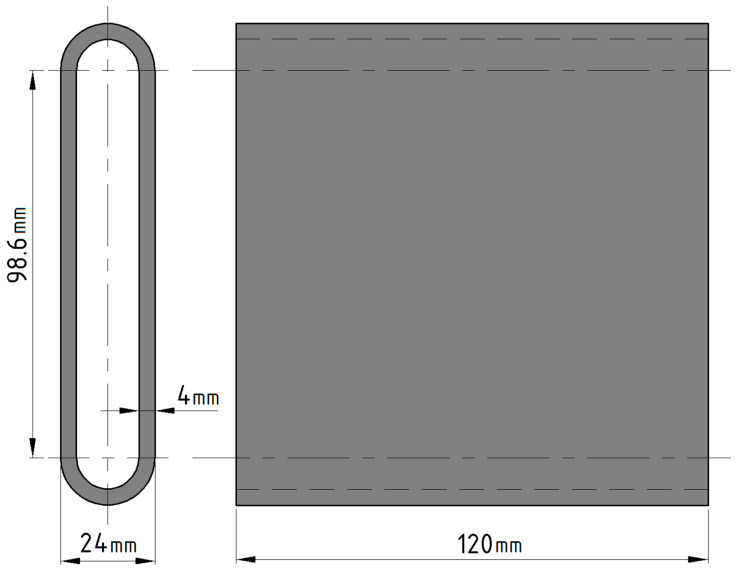
260 mm length loop specimen dimensions.

Using direct laser metal sintering it is possible to produce objects and models with high precision. However, the disadvantage of this manufacturing process is the production time and costs involved. For small and medium volume parts, the production time is around 6–12 h or even more [26]. Gibson et al. [3] categorized the cost of additive layer manufacturing into four main categories (Equation (1)): machine-, production-, material and operation costs. Print speed determines the production time, which has a significant impact on production costs. Based on these, the other metal printing processes are compared with CMT in focus of productivity [3]. One of the essential elements of the cost of production is the printing time. The layer thickness, laser scan speed and orientation are determinative factor of fabricating time [27]. In our study, the production speed is determined by the CMT toolhead movement velocity. For the 260 mm specimen (Figure 16), the calculations were performed based on process optimization of Verma et al. and using the guidelines of Caligano et al. [26,28]. This demonstrates the productivity advantage of the CMT AM process over other metal print technologies (Table 9).

The manufacturing cost of a 260 mm specimen selected from Table 4 as an example in the aspect of the known production times and costs for each additive manufacturing technology (Table 1) are shown in Table 10 and calculated according to:

(1) $$C_{total}=C_{P}+C_{O}+C_{M}+C_{L}$$

where

*C_P_*: equipment purchase cost, calculated according to Equation (2).

*C_O_*: machine Operation cost, calculated according to Equation (3).

*C_M_*: material cost, from Table 11.

*C_L_*: labor cost, based on the hourly average salary of a technician in Hungary (15 EUR).

Calculation of machine and operation costs are based on the recommendation of Gibson et al. [3]. the machine purchase cost for one specimen, assuming a 3-year running time of the equipment, is:

(2) $$C_{P}=\frac{Purchase\;price\times T_{b}}{0.95\times 24\times 365\times 3};$$

where *T_b_* is the building time of one specimen.

The machine operation cost for one specimen is:

(3) $$C_{O}=T_{b}\times \frac{Annual\;maintenance\;cost+Annual\;overhead\;cost}{0.95\times 24\times 365};$$

The total material cost of a specimen is the product of the mass of the required material in kilograms and the cost per kilograms of the given base material for each manufacturing technology, as shown in Table 11.

Labor cost is the product of hourly salary and the required active labor time of the technician. Labor time includes the time required for setting up and operating the equipment and specific post-production times like removing the specimen from the base plate in case of CMT-based additive manufacturing or de-dusting the chamber in case of powder-based technologies.

## 5. Conclusions

The results demonstrate that the productivity—in terms of speed—of the additive manufacturing technology based on CMT is highly superior to other solutions. It is capable of deposition of large amount of material relatively quickly, with a high toolhead moving speed, while maintaining the mechanical properties of the base material, which is an important observation regarding CMT-based 3D printing. However, the surfaces are needed to be machined after the additive manufacturing process to achieve the eventually required tolerances, therefore, we have to emphasize that it is not capable for producing finished metal parts, like the DMLS technology, but it can used well in prototyping and modelling. To maintain proper fusion of layers and precise geometry, the sets of settings should be used that were found stabile during the experiments (Table 4). The first four layers require a higher heat input and the toolhead movement speed has to be between 65 mm/s and 75 mm/s to maintain arc stability.

The economical calculations demonstrate that using CMT AM, production cost is much less than of DMLS or EBM technologies. This is mainly the result of the lower investment and maintenance costs and the drastically higher productivity. It is important to note, if further processing such as machining is needed, their costs and time must be calculated as well. In this study, optimal settings have been found, which allow hollow parts to be built with short manufacturing time. Based on experiments heat input and welding speed highly define wall-geometry. The seams visibly flatten in specimens with shorter loop lengths as in this case the cooling time of each layer is shorter. As shown in Figure 9, above 60 mm height, the seam width is relatively constant. Based on our examination it can be stated that CMT additive manufacturing has significantly shorter production time, lower operating cost and investment cost compared to other metal printing methods. The disadvantage of the studied process is a lower precision of the products; however, it can be improved by machining. In addition, we have to note that this method is not suitable for finished part production directly. In further experiments, it would be advantageous to research a combined manufacturing procedure of CMT AM and CNC machining. This might provide the required surface quality while maintaining the short production time.

## Figures and Tables

**Table 1:** Comparison of AM equipment main prices and maintenance costs. Adapted from: [7].

Costs and Properties	EBM	DMLS	CMT AM
Main Machine Price (MMP)	925,000 EUR	550,000 EUR	16,500 EUR
Auxiliary Equipment Price	Included in MMP	65,000 EUR	2200 EUR
Annual Maintenance Cost	42,000 EUR	29,000 EUR	890 EUR
Annual Overhead Cost	46,200 EUR	27,300 EUR	810 EUR
Floorspace	16.8 m^2^	13.2 m^2^	4 m^2^
Resolution	0.28 mm^2^	0.24 mm^2^	0.984 mm^2^
Volume flow	3.61 mm^3^/s	4.23 mm^3^/s	74.6 mm^3^/s

EBM, electron beam technology; DMLS, direct metal laser sintering; CMT AM, cold metal transfer additive manufacturing.

**Table 2:** Chemical composition of Ø1.2 mm Böhler S-Al Mg 4.5 Mn welding wire (wt%).

Si	Fe	Cu	Mn	Mg	Cr	Zn	Ti
Max.0.4	Max.0.1	Max.0.1	0.4–1.0	4.3–5.2	0.05–0.25	Max.0.25	Max.0.15

**Table 3:** Welding parameters of overlaid weld layers.

Welded Layers	I [A]	U [V]	v_wire_ [m/min]	Process
1	90	16.9	5.2	MIX
2	110	14.2	7.6	CMT
3	90	12.4	6.2	CMT
4	69	11.5	4.7	CMT
5–300	59	11.3	4.0	CMT

**Table 4:** Combination of stabile settings of CMT additive manufacturing.

Set Id.	t [s]	v_welding_ [mm/s]	Length [mm]
1	4	65	260
2	6	65	390
4	4	75	300
5	6	75	450

**Table 5:** Mean chemical composition of welding wire by SEM analysis (wt%). The results are averages measured at three points. The results have to be considered to be approximate.

	C	O	Mn	Mg	Al
Measured value	3.45 ± 0.39	1.56 ± 0.04	0.45 ± 0.15	3.82 ± 0.02	90.73 ± 0.56
Cor. value	---	---	0.47 ± 0.162	4.01 ± 0.018	95.26 ± 0.592

**Table 6:** Heat input and volume flow for each set of settings.

Set Identity	Specific Heat Input * [J/mm]	Volume Flow [mm^3^/s]
1	12.715	75.44
2	10.684	74.12
4	9.410	75.30
5	11.061	73.50

* Acquired from CMT welding equipment divided by the total weld length.

**Table 7:** Tensile strength of AlMg4.5Mn0.7 3D printed specimens manufactured parallel with the welding direction.

Set Id.	Average R_m_	Tensile Strength R_m_ [MPa]										
1.	259 ± 16	216	247	260	267	267	272	270	271	264	261	259
2.	243 ± 29	257	192	210	250	186	214	250	256	258	258	265
4.	270 ± 4	273	273	262	264	276	270	270	268	272	273	268
5.	264 ± 11	260	276	265	257	292	254	264	251	270	264	274

**Table 8:** Tensile strength of AlMg4.5Mn0.7 3D printed specimens manufactured perpendicular to the welding direction.

Set Id.	Average R_m_	Tensile Strength R_m_ [MPa]										
1.	157 ± 14	169	164	174	164	138	174	156	155	162	135	141
2.	246 ± 17	240	265	219	251	238	244	255	271	242	221	260
4.	241 ± 9	243	234	241	246	238	242	232	235	235	239	266
5.	236 ± 25	259	265	258	226	209	232	246	249	228	179	246

**Table 9:** Estimated cost of aluminium additive manufacturing.

Process	Layer		Powder	Wire	Building
	Count	Thickness	Diam. Range	Diameter	Time (T_b_)
CMT AM	300	0.40 mm	n/a	1.2 mm	0.54 h
DMLS	2000	0.06 mm	5–72 µm	n/a	9.60 h
EBM	1714	0.07 mm	35–105 µm	n/a	8.20 h

**Table 10:** Estimated cost of 260 mm length loop specimens.

Technology	*C_P_* [EUR]	*C_O_* [EUR]	*C_M_* [EUR]	*C_L_* [EUR]	*C_total_* [EUR]
CMT AM	0.40	0.11	1.92	9.11	11.55
DMLS	236.48	64.95	14.4	147.28	463.1
EBM	303.81	86.91	14.4	125.8	530.92

**Table 11:** Total estimated material cost of a 260 mm specimen.

Welding Material	Specific Material Cost [EUR/kg]	Specimen Mass [kg]	Specimen Material Cost [EUR]
Al wire	4	0.48	1.92
Al powder	30	0.48	14.40
